# Supplementary material for: DeepDOF-SE: affordable deep-learning microscopy platform for slide-free histology
Source: Nat Commun. 2024 Apr 5;15:2935. doi: 10.1038/s41467-024-47065-2 (PMC10997797; doi:10.1038/s41467-024-47065-2)
Supplement: Supplementary file 1 — Supplementary information [file 41467_2024_47065_MOESM1_ESM.pdf]

Supplementary Information

DeepDOF-SE: affordable deep-learning microscopy platform for slide-free histology

Supplementary Note 1: Tissue processing workflow and cost estimation

Supplementary Figure 1 shows the typical workflow, required equipment, and estimated cost for frozen section H&E and slide-free histology using DeepDOF-SE. Once the tissue is resected, a pathologist cuts the specimen into 3-4 mm thick slices using a scalpel to examine the cross-section area. Suspicious slices will be sent for downstream processing.

For frozen section processing, a cryostat is used to quickly freeze the tissue and section it into thin 5-10 μm slices. A motorized cryostat (for example, CryoStar™ NX70 or Leica CM 3050 S) costs from $70,000 to $100,000. In permanent H&E, the tissue is first formalin fixed and paraffin embedded (FFPE) before thin sectioning, requiring over 24 hours for processing. An automated rotary microtome used in FFPE H&E costs over $40,000 (for example, Tissue-Tek AutoSection). After the thin tissue slice is mounted onto glass slides, they are stained in batches in a slide stainer. An automated slide stainer such as HistoTek SL costs around $35,000 while a high throughput slide stainer costs as much as $60,000 (for example, ShurStain). After staining, the slide is either manually scanned by the pathologist under a conventional light microscope or scanned and digitized using a slide scanner. The cost of most commercial whole slide imaging devices ranges from $30,000 to $250,000 even though an effective low-cost build can be achieved for $2,500[1].

In contrast, DeepDOF-SE only requires simple staining after the tissue is bread loafed into thick sections. The specimen cross section is stained with DAPI and Rhodamine B (<$5/sample). The stained tissue is imaged using DeepDOF-SE (<$7,000, see Supplementary Table 1), and the image is processed and stored on a $2,000 workstation. DeepDOF-SE costs only a fraction of that of other more complex systems designed for slide-free histology, such as the confocal microscope (Vivascope, $100,000) or full field OCT (LLTech, >$100,000)[2].

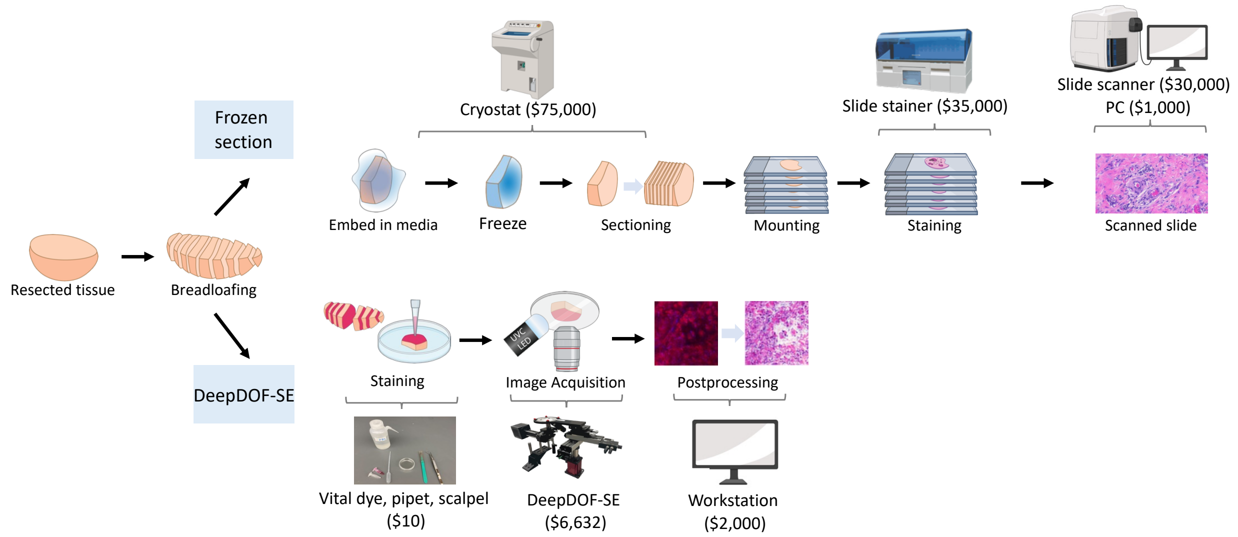

Supplementary Figure 1: Workflow and associated equipment for frozen section histology vs. slide-free histology using DeepDOF-SE. The slide-free DeepDOF-SE requires less equipment, simplified procedures, and shorter time, altogether leading to ease of use and reduced cost. Figure created with Biorender.com.

Supplementary Table 1: DeepDOF-SE building cost breakdown

Item	Model	Cost	Count	Total
PC with GPU	built from parts	2000	1	2000
Motor	X-LHM100A	1233	2	2466
Camera	Tucsen FL20	2200	1	2200
LED	Thorlabs M275L4	400	1	400
Objective	RMS4X-PF	611	1	611
Dual-band emission filter	Chroma 59003m	350	1	350
Sapphire window	KnightOptical WHF5053	103	1	103
Tube lens	Thorlabs AC254-150-A	87	1	87
Phase mask	fabricated in house, cost amortized	15	1	15
Other opto-mechanical items (cage plate etc.)	Thorlabs	400	1	400
Microscope cost				6632
Total cost (with PC)				8632

Supplementary Table 1: Detailed cost of parts used to build DeepDOF-SE at the time of purchase. The microscope itself costs $6632 while the workstation (including the GPU) costs $2000.

Supplementary Note 2: Depth-of-field range and tissue irregularity characterization

The targeted DOF of 200 µm was determined based on irregularities in the surface of thick tissue slices cut with a surgical scalpel. As shown in Supplementary Figure 1, per standard of care, a pathologist cuts a resected surgical specimen into 3-4 mm thick slices (bread loafing). While irregularities on the scalpel-cut surfaces exceed DOF of a conventional microscope with

subcellular resolution, DeepDOF-SE is designed to directly image scalpel-cut surfaces in Supplementary Figure 1 without need for refocusing. Scalpel-cut tissue surfaces were previously reported to have surface irregularities of up to 200 µm in height[3]. In our study, we also characterized the surface profile of porcine tongue slices cut with a pathology scalpel. With manual refocusing, we recorded the axial range of surface irregularities in 200 FOVs (each measured 0.87x0.65 mm^2) from four different tissue slices, and our results shown in Supplementary Figure 2 are consistent with previously reported results[3].

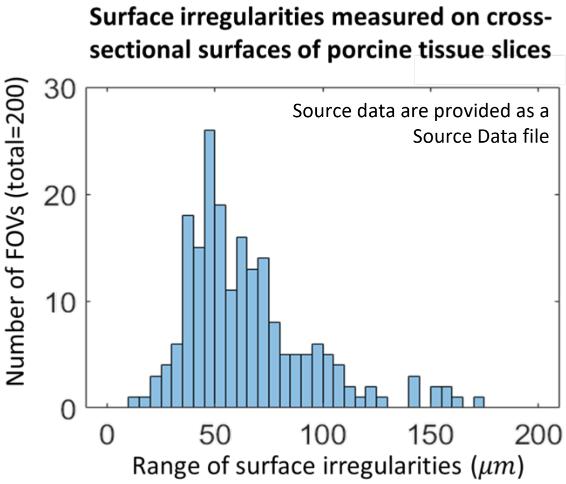

Supplementary Figure 2: Histogram of surface irregularities of porcine tongue in 200 FOVs. Fresh porcine tongue was acquired from an abattoir, sliced with a pathology scalpel, stained with a vital dye (proflavine), and placed on a glass slide. The cross-sectional surfaces of four slices were examined using a Zeiss Axiovert 200 microscope (10X, NA 0.25). For each FOV, the tissue is scanned in the z-axis until every region has been in focus once, then the z range is recorded as the range of surface irregularity.

Supplementary Note 3: Objective lens design choice

We specifically designed DeepDOF-SE with a 4X, 0.13 NA objective to provide a slide-free histology platform for use in low-resource settings to support immediate biopsy assessment and/or rapid intraoperative assessment of margin status.

This use case is consistent with a recent study by Brown and colleagues, in which the pathologist's use of 2X, 4X, 10X, and 20X objectives was monitored digitally during diagnostic review of H&E stained slides from a radical prostatectomy case[4]. The pathologist used the 2X objective to examine 95% of the area. In comparison, the 10X objective was used to examine only 2% of the specimen area and the 20X objective was used to examine less than 0.15%. Similarly, in Mohs micrographic surgery, skin cancers are removed in stages until histologic examination of frozen sections confirms negative tumor margins[5]. Typically, frozen sections are examined with 1X-2X magnification[6,7] which is adequate to assess margin status. A higher NA (for example, at 10X, 0.30 NA or higher) is needed to resolve some sub-cellular features (e.g., vascular and perineural invasion, and for phenotypic characterization of tumor cells in undifferentiated malignancies)[8]; however, this is typically not necessary for diagnosis of neoplasia.

Moreover, there are important tradeoffs associated with increasing the NA, including a smaller field of view and reduced depth of field. As previously shown by Baek et al., there exists a fundamental tradeoff between the transmission efficiency and depth invariance of an imaging system that has a modulated pupil function[9]. We simulated the performance of DeepDOF-SE with a 10x 0.30 NA objective. Supplementary Figure 3 below shows the Root Mean Square Error (RMSE) curve of simulated reconstruction (averaged over both fluorescence channels and 8000 images) across 21 defocus depths. Compared to the conventional baseline with the same objective lens, the DOF was significantly expanded from 7 microns to 40 microns (5.4x increase). However, this DOF is still far from the 200 microns required for imaging scalpel-cut irregular tissue surfaces. In this 40-micron DOF range, higher RMSE and decreased imaging performance were observed in defocus ranges of +/- 15 – 20 microns, making it challenging to resolve features in a target 200 μm DOF range.

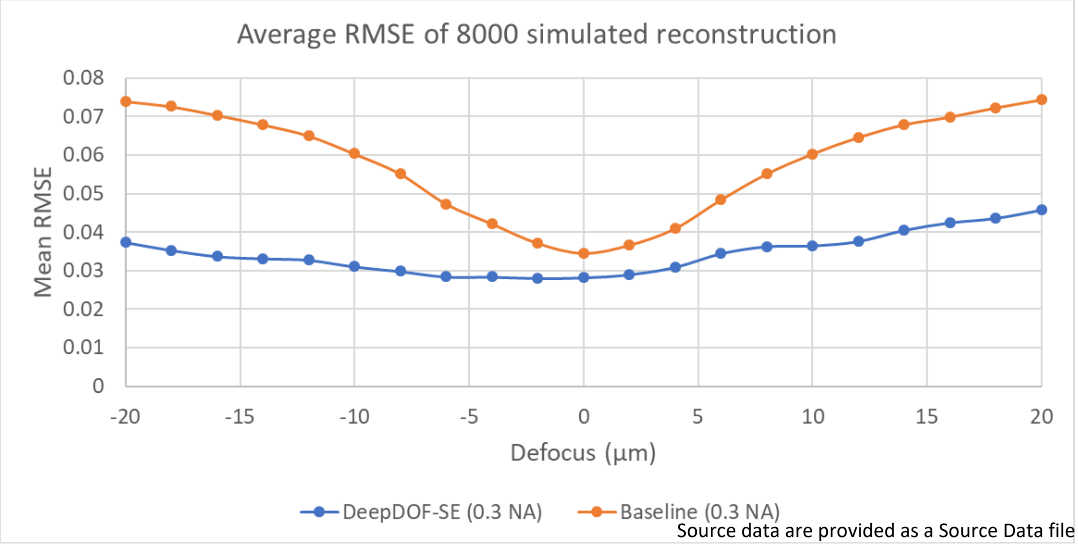

Source data are provided as a Source Data file

Supplementary Figure 3: Average mean root square error (RMSE) of 8000 simulated images (2 fluorescence channels at 473 nm and 640 nm, respectively) of a baseline conventional microscope (10x, 0.3 NA) and DeepDOF-SE (10x, 0.3 NA). The error is calculated by comparing the captured images, or in DeepDOF-SE's case, the U-Net reconstruction with the ground truth all-in-focus images. The DeepDOF-SE model here is optimized end-to-end for a 40 μm depth-of-field, a 5.4x increase from the 7.4 μm original depth-of-field.

Supplementary Note 4: Achromaticity of the DeepDOF-SE Design

To demonstrate chromatic aberration between the two fluorescence channels, we imaged a frozen section of a mouse tongue stained with Rhodamine B and DAPI using a conventional microscope at two axial planes that are 50 μm apart. As shown in Supplementary Figure 4, the image in Rhodamine channel is in focus at axial plane 1, while the image in DAPI channel is in focus at axial plane 2. In contrast, in Figure 4 DeepDOF-SE images in both channels are consistently in focus across the entire DOF range.

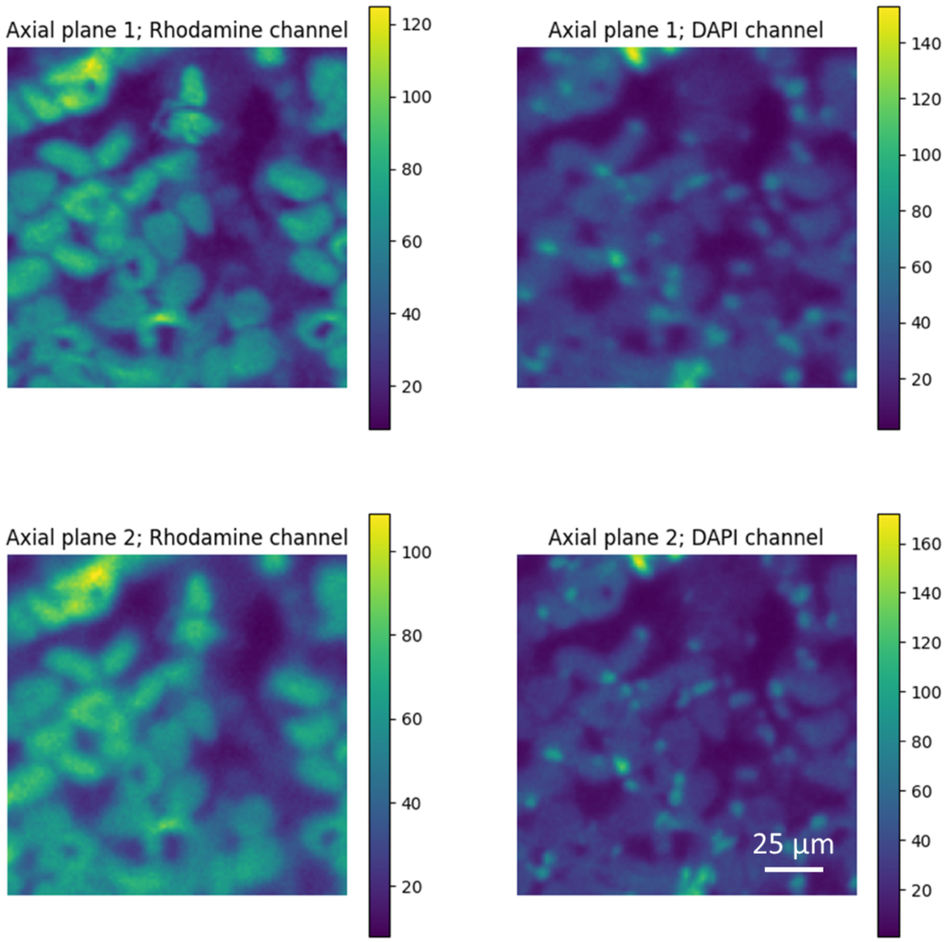

Supplementary Figure 4: Images of mouse tongue frozen slides acquired using a conventional microscope, showing chromatic aberrations observed in two fluorescence channels at two axial planes (axial planes 1 and 2 are 50 μm apart). The image in Rhodamine channel is in focus at axial plane 1, while the image in DAPI channel is in focus at axial plane 2.

While the end-to-end framework described in Figure 2 is optimized for DAPI and Rhodamine B fluorescence channels in the current work, we also explored the feasibility of using the end-to-end framework to achieve EDOF imaging in a broadband spectral range. Results in Supplementary Figure 5 demonstrate that the DeepDOF-SE design is highly achromatic, showing that the system is widely compatible with other vital dyes and combinations of contrast agents for multiplexed imaging in multiple fluorescence channels. Here, spectral ranges in the R (640 nm), G (532 nm), and B (473 nm) channels are chosen based on the camera sensor filter.

To evaluate the achromaticity and depth-of-field of the optimized design including the learned mask and reconstruction networks, we simulated the modulated transfer function (MTF) of the system. For each of the 21 discrete defocus depths, a ground truth USAF resolution target is used to simulate an RGB sensor image. The simulated sensor image is then reconstructed by the U-Nets. For each color channel in the reconstructed images, the contrast of 7 line-pair (lp)

groups ranging from 87 lp/mm (11.5 μm line width) to 347 lp/mm (2.9 μm line width) are calculated as follows:

$$contrast(img\_lp) = \frac{max(img_{lp}) - min(img_{lp})}{max(img_{lp}) + min(img_{lp})}$$

$$contrast\_(grp\ i) = \frac{horizontal\ contrast_{grp\ i} + vertial\ contrast_{grp\ i}}{2}$$

The MTF plots for selected defocus depths are displayed in Supplementary Figure 5. The close proximity of MTFs in 3 color channels shows that the end-to-end optimized system is highly achromatic. All 3 color channels achieved high contrast in the majority of frequency ranges within the DOF. In contrast, the conventional microscope's MTF shows rapid decrease in area under the MTF curve as the defocus increases. The effects of chromatic aberration can also be observed in the separation of MTF curves. However, we note that the forward optical model does not fully capture the aberration caused by the objective and tube lens of the experimental system.

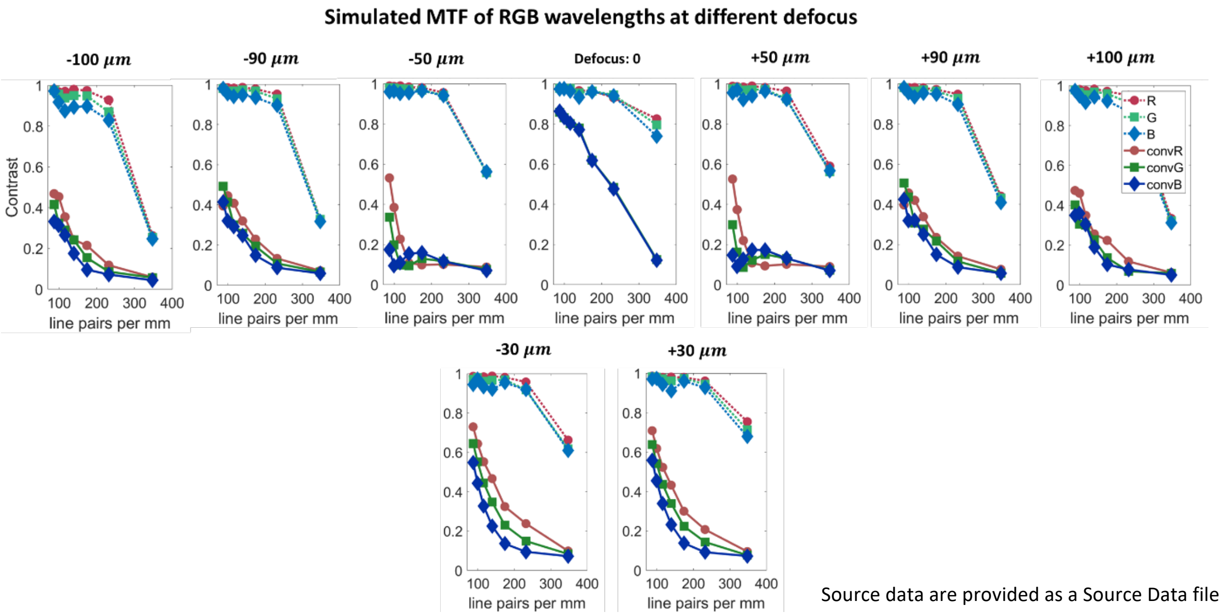

Source data are provided as a Source Data file

Supplementary Figure 5: Simulated Modulated transfer function (MTF) of the system at 7 selected defocus depths for DeepDOF-SE's R (640 nm), G (532 nm), and B (473 nm) wavelengths and a conventional baseline (4x 0.13 NA)'s convR (640 nm), convG (532 nm), convB (473 nm). The similarity among the MTFs of the DeepDOF-SE at the 3 wavelengths shows that end-to-end optimized system is highly achromatic and capable of retaining high frequency information in the designed depth-of-field. The conventional microscope not only suffers from defocus aberrations, but also chromatic aberrations.

Supplementary Note 5: MS-SSIM calculation for frozen section slide imaging with defocus

In Figure 4, we used the image at 0 μm defocus for the respective microscopes as the ground truth when comparing the MS-SSIM across different depths. The MS-SSIM between the conventional and DeepDOF-SE at 0 μm defocus are as follows: Colon: 0.9039; esophagus: 0.8731; liver: 0.9001. As shown in Supplementary Figure 6, the conventional image has a higher

noise level than DeepDOF-SE, which contributes to the discrepancy in the MS-SSIM score at 0 μm defocus. This is also consistent with our previous observation that the U-Net in DeepDOF-SE was trained with added noise and has been shown to have a denoising effect.

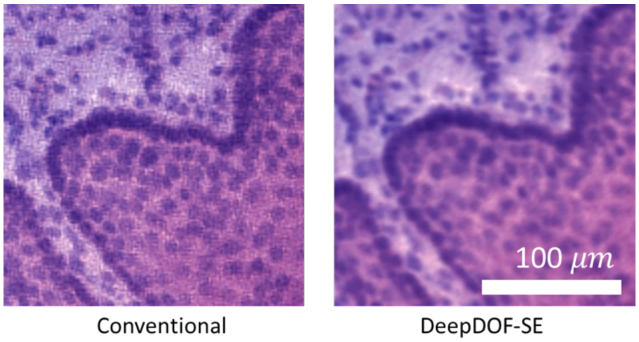

Supplementary Figure 6: Frozen section slide of human esophagus imaged by conventional ultra-violet excitation microscopy (left) and DeepDOF-SE (right) at 0-micron defocus. The MS-SSIM between the two field-of-view is 0.8731. The proposed DeepDOF-SE is able to resolve the nuclei as well as the in-focus conventional while appearing less noisy.

Supplementary Note 6: Regularization effects of the 2-step training in CycleGAN

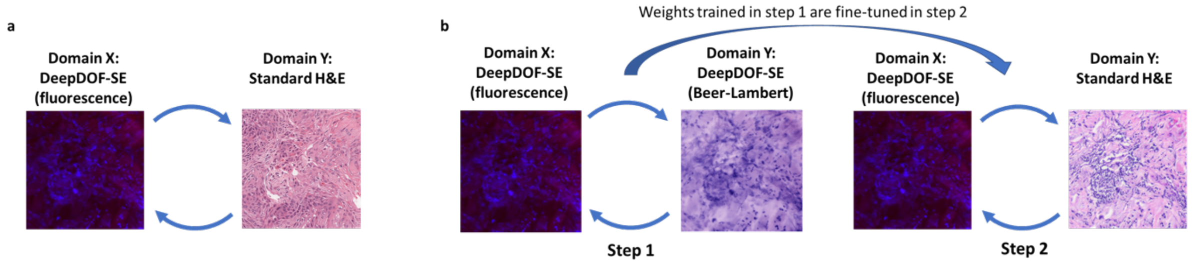

*Supplementary Figure 7: Two training schemes for the CycleGAN for virtual H&E staining. **a**. 1-step unsupervised training that directly translates fluorescence DeepDOF-SE images to standard H&E. **b.** 2-step semi-supervised training: in step 1, paired DeepDOF-SE fluorescence and DeepDOF-SE Beer-Lambert virtual H&E is used to train the CycleGAN (supervised); in step 2, the same CycleGAN weights are fine-tuned by replacing the DeepDOF-SE Beer-Lambert virtual H&E with the standard H&E (unsupervised). Rhodamine B channel for the fluorescence image brightened for display.*

In order to train a CycleGAN for virtual H&E training, there are two possible models. The first model, as presented in Supplementary Figure 7a, directly translates DeepDOF-SE fluorescence reconstruction into the domain of standard H&E slides. While the input requires no preprocessing, CycleGAN fails to learn the color transformation with cycle consistency loss alone. The brighter nuclei in the fluorescence image erroneously show up as white empty space in the virtual H&E. Similarly, the black background in the fluorescence input is mistaken as dark nuclei in the GAN output.

Using a semi-supervised method, we show that CycleGAN is in fact capable of learning the color transformation. Supplementary Figure 7b describes the 2-step training process. In step 1, CycleGAN is trained in a supervised fashion with paired fluorescence and Beer-Lambert virtual staining images. This step forces the network to learn the color transformation. In step 2, the same network is fine-tuned by replacing the DeepDOF-SE Beer-Lambert virtual H&E with the standard H&E. Although step 2 is unsupervised, CycleGAN still produces correct mapping since

the network has already learned the color transformation in step 1. The final trained CycleGAN can directly map DeepDOF-SE fluorescence images to virtual H&E in a single feedforward step.

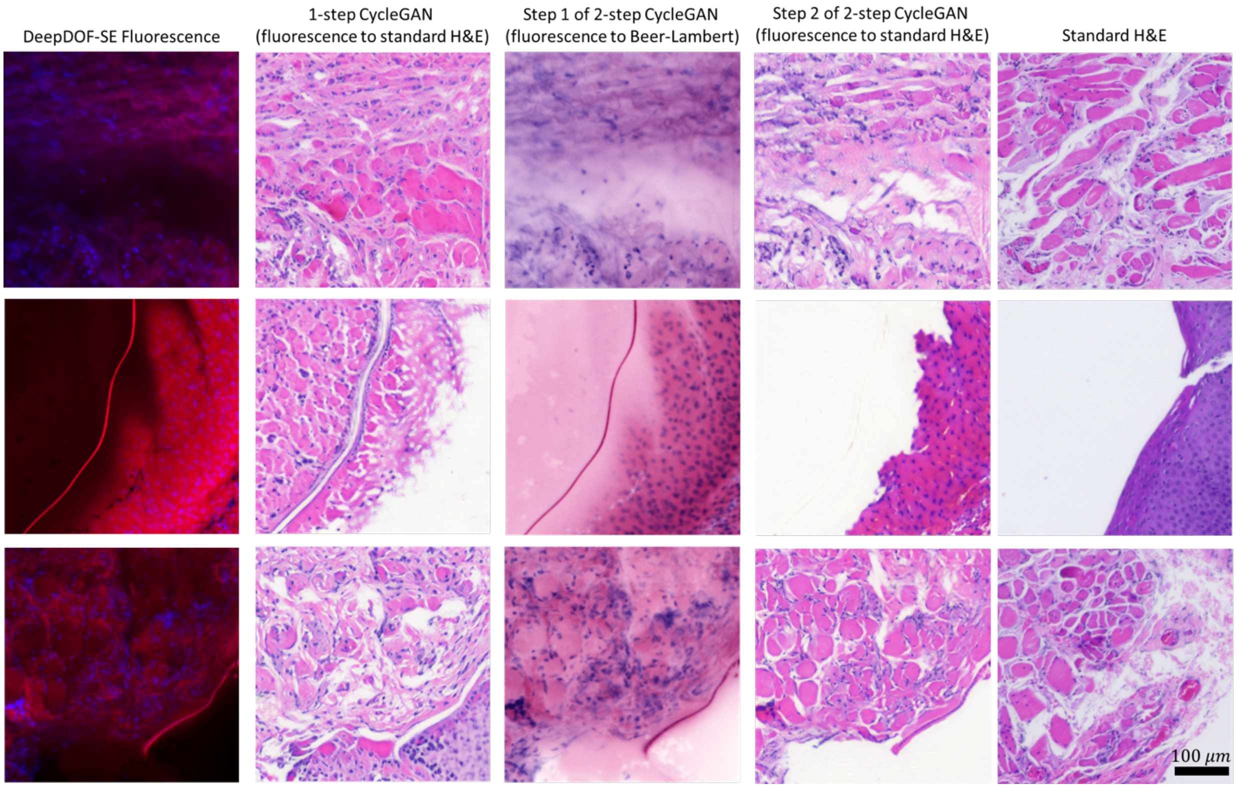

Supplementary Figure 8: Comparison of 1-step CycleGAN (Supplementary Figure 7a) versus the reported 2-step CycleGAN (Supplementary Figure 7b). The bright blue nuclei in the fluorescence image are incorrectly translated to white space in the 1-step CycleGAN (column 2 from the left), as the network fails to learn the color translation. Step 1 of our 2-step training scheme forces the CycleGAN to learn the color transform (column 3 from the left). After the 2-step training, the CycleGAN is able to translate with both the color and style of the standard H&E correctly (column 4 from the left).

Supplementary Note 7: Quantitative comparison of Beer-Lambert, CycleGAN virtually stained tissue, and standard H&E

To validate the performance of CycleGAN virtual staining, we quantitatively compared the CycleGAN staining to standard H&E staining. Mouse tongue frozen section slides were first imaged using DeepDOF-SE, and they were sent to a pathology laboratory for conventional H&E processing. We empirically validated that the fluorescence staining used for DeepDOF-SE does not affect the downstream H&E processing. Supplementary Figures 9 shows the Beer-Lamber stained, CycleGAN stained mouse tongue slide, and the corresponding gold standard H&E scan. While some color differences are observed, the nuclei thresholding results show that the location and shape of the nuclei in the CycleGAN virtual staining images closely resemble those in the conventional H&E images.

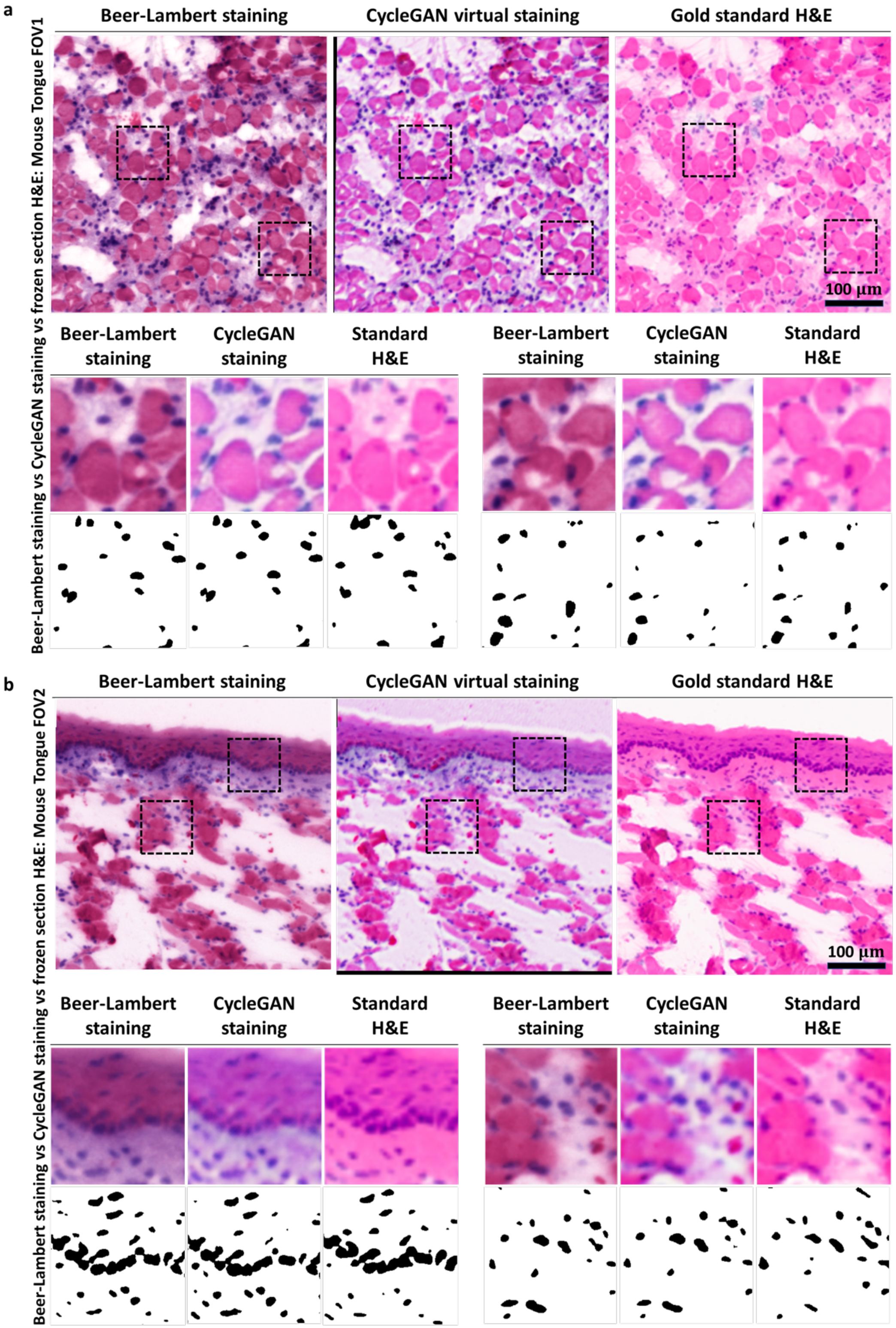

Supplementary Figure 9: Frozen section mouse tongue slide imaged using DeepDOF-SE with CycleGAN staining, Beer-Lambert-law based staining, and corresponding standard H&E staining of the same slide. The large FOVs are 504 x 504 µm, and correspond to the FOV1 and FOV2 in Supplementary Table 2 respectively. Insets show magnified regions of interests and their corresponding nuclei thresholding results.

We further quantified the results by comparing the nuclear count and mean nuclear area in the virtually stained images (CycleGAN and Beer-Lambert-law based method) and the conventional H&E images shown in Supplementary Figure 9. The virtually stained images were warped to align with the standard H&E. We used the open-source software "Cell Profiler"[10] to automatically segment and count the cell nuclei. Four 504 x 504 μm FOVs of the mouse tongue frozen section, including 2 shown in Supplementary Figure 9, are selected and the results are displayed in Supplementary Table 2. The close match of both metrics between the CycleGAN staining and standard H&E demonstrates that the CycleGAN-based virtual staining provides histology information of diagnostic importance without generating undesired artifacts. The small differences in the nuclear count and nuclear area can be ascribed to the staining color differences observed in Supplementary Figure 9, errors in automated segmentation, and discarded nuclei near the FOV edges. When comparing the mean nuclear area, CycleGAN virtual staining provides a closer match than Beer-Lambert-law based method, potentially due to the closer color to the H&E resulting in better channel demixing for nuclei segmentation.

	**Nuclear Count**			**Mean Nuclear Area (μm^2)**		
	**Beer-Lambert**	**CycleGAN H&E**	**Standard H&E**	**Beer-Lambert**	**CycleGAN H&E**	**Standard H&E**
**FOV 1**	**471**	**465**	**466**	**40.40**	**31.93**	**27.84**
**FOV 2**	**424**	**433**	**426**	**36.02**	**31.00**	**28.11**
**FOV 3**	**510**	**548**	**567**	**39.60**	**37.27**	**35.35**
**FOV 4**	**448**	**431**	**448**	**34.31**	**31.83**	**28.36**

Supplementary Table 2 Nuclei count and mean nuclear area comparison between CycleGAN virtually stained and standard H&E images of mouse tongue frozen section. Each FOV is 504 x 504 μm. FOV1 and FOV2 are shown in Figure 6c and Supplementary Figure 9. Source data are provided as a Source Data file.

In the case of frozen section slide virtual staining, the algorithm only needs to perform color transformation since the captured image already contains slide-based features. When comparing Beer-Lambert-based virtual staining and CycleGAN staining of fresh tissue (Supplementary Figure 10), it can be observed that the CycleGAN generates images that more closely resemble physically stained H&E slides. For instance, the white space between muscle fibers is present in both the CycleGAN virtual staining and the FFPE H&E, but not the Beer-Lambert-based staining. By learning the style of physically stained H&E slides, CycleGAN virtual staining has higher contrast and more closely resembles FFPE H&E used by pathologists.

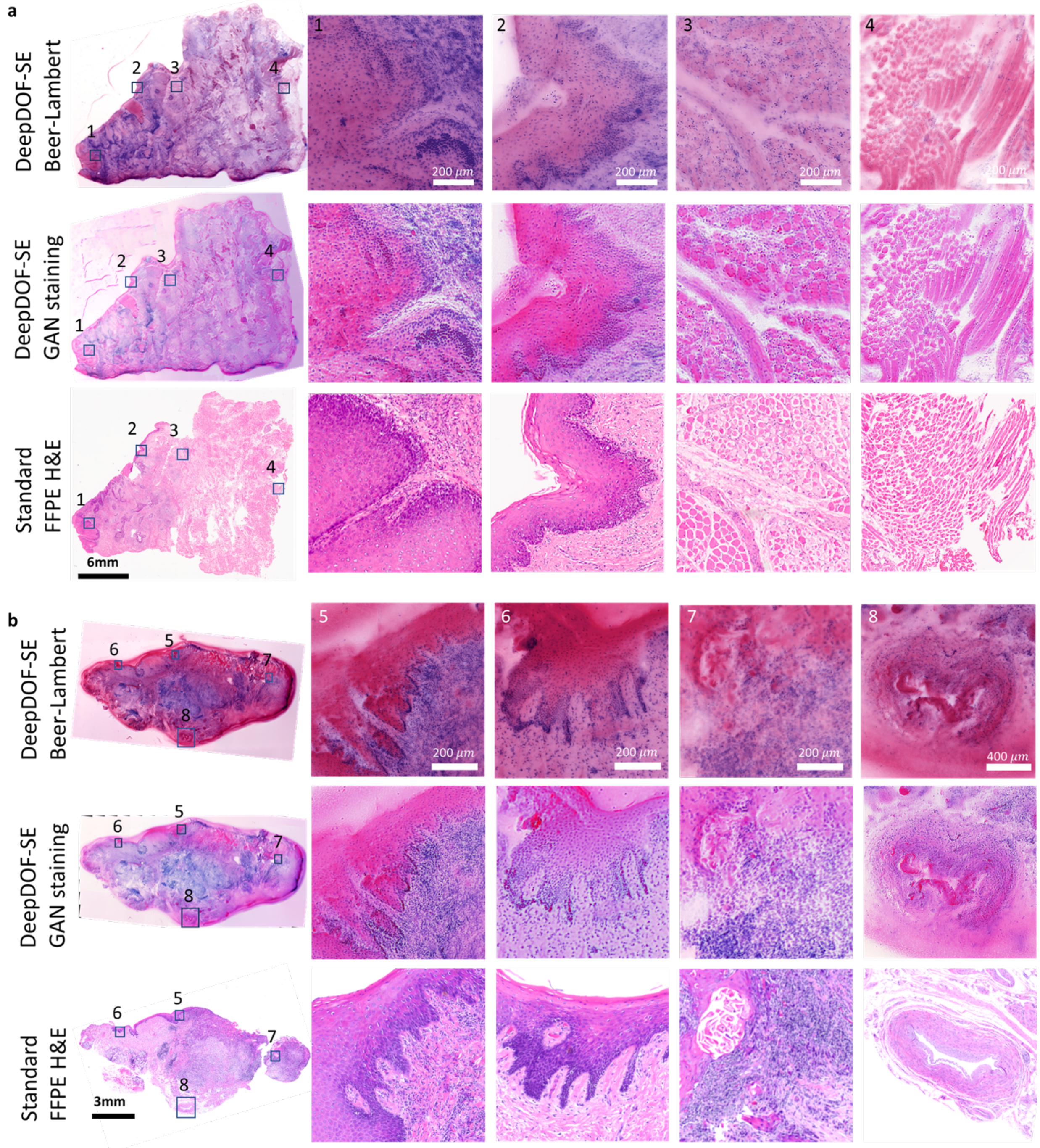

Supplementary Figure 10: same figure as Figure 7 in the main text, replacing DeepDOF-SE fluorescence with DeepDOF-SE Beer-Lambert virtual staining to show the comparison between Beer-Lambert H&E and CycleGAN H&E. In both samples, the CycleGAN virtual H&E's color appears closer to the standard FFPE H&E. The CycleGAN H&E also learns the style of the thin cut slide where regions with no tissue appear white.

We conducted a blinded review of histological features to further assess the diagnostic value of DeepDOF-SE images quantitatively. In this pilot evaluation, 20 DeepDOF-SE fluorescence images of fresh oral tumor resections, each containing varied histological features in a 2mm x 2mm FOV, were obtained. These fluorescence images were processed using the Beer-Lambert-law-based

method and with CycleGAN staining, resulting in a total of 40 images each covering an FOV of 4 mm^2. De-identified images were presented in randomized order to two expert pathologists who were asked to evaluate each image and assess the degree to which image quality was sufficient for 1) identification of architecture and normal structures, and 2) diagnosis of neoplasia. For each metric, three quality scores were used: 1=poor image quality, not sufficient for diagnosis, 2=moderate image quality but sufficient for diagnosis, and 3=good image quality, sufficient for diagnosis.

Supplementary Figure 11 shows the mean image quality scores for the two image types; the mean image quality score was higher for images stained using CycleGAN for both pathologists. A Wilcoxon signed-rank test showed a significant difference ($Z = -2.55$, $p<0.05$ for both metrics) between scores given for Beer-Lambert-law based images and CycleGAN images.

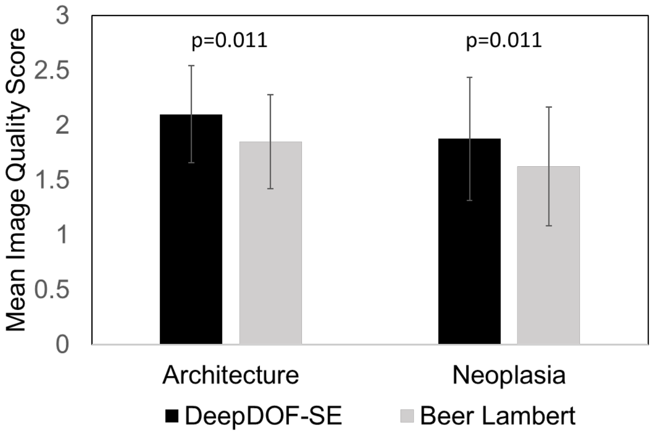

Source data are provided as a Source Data file

Supplementary Figure 11: Mean image quality scores for images stained with CycleGAN (DeepDOF-SE) and Beer-Lambert method, showing a higher score using CycleGAN. A Wilcoxon signed-rank test showed a significant difference ($Z = -2.55$, $p<0.05$ for evaluation of architecture and neoplasia) between the two methods. (n=20).

Supplementary Note 8: CycleGAN virtual staining applied to other tissue types

It is critical to validate CycleGAN performance using data not seen in the training set. Following CycleGAN training with images of fresh human oral tumor, we evaluated model performance using images from three different tissue types. Other than the mouse tongue tissue and the fresh human oral surgical samples, we imaged frozen sections of mouse esophagus. Supplementary Figure 12 shows an image of a mouse esophagus stained virtually using the CycleGAN algorithm. The image clearly shows the esophageal architecture with epithelium and surrounding connective tissue and muscle. As shown in selected ROIs, nuclei in the epithelial layer and connective tissue in the lamina propria in ROIs 1 and 2, as well as muscle fibers in ROIs 3 and 4, are visualized in the virtually stained DeepDOF-SE images. Since CycleGAN virtual staining was trained using oral tissue images, we expect improved staining performance can be achieved in future work by training the model with an expanded image library of specific tissue types.

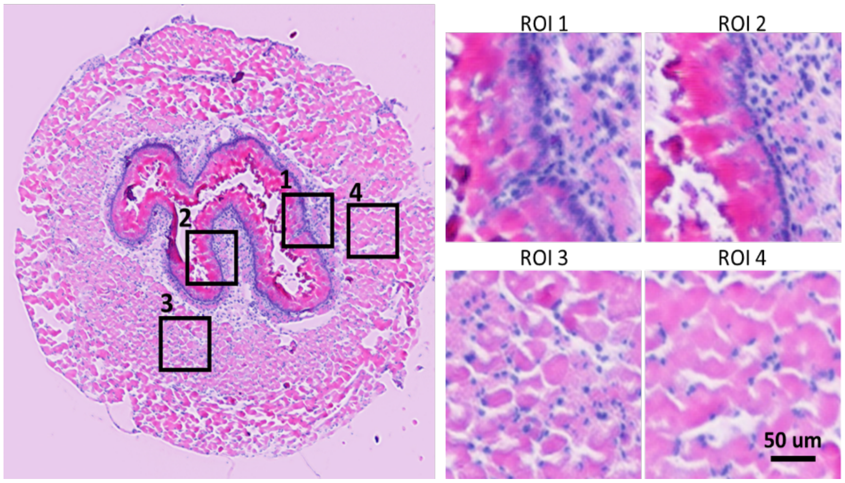

Supplementary Figure 12: Virtually stained image of a frozen slide of mouse esophagus. Nuclei in the epithelial layer and connective tissue in the lamina propria in ROIs 1 and 2, as well as muscle fibers in ROIs 3 and 4, are visualized in the DeepDOF-SE image.

Supplementary Note 9: Exceptional cases in CycleGAN results

While the CycleGAN is capable of virtually staining various tissue types such as the layered epithelium and muscle fibers, we observed some differences between CycleGAN virtually stained images and H&E images, due to inherent differences in staining mechanisms and sample processing. In these exceptional cases, CycleGAN stains these areas similar to the analytical Beer-Lambert method, preserving features in the fluorescence images. For instance, adipose cells usually have a web-like appearance in the conventional slide-based H&E due to mechanical sectioning and loss of lipids during H&E processing. Since DeepDOF-SE images fresh tissue, adipose cells appear intact in the fluorescence images. Supplementary Figure 13 top row shows the round sphere-shaped adipose cells in the fluorescence, Beer-Lambert virtual staining, and CycleGAN virtual staining images, even though the same area shows a honeycomb architecture in the corresponding standard H&E images. Despite the visual differences, the intact adipose cells in CycleGAN stained images possess a distinct look and are readily discernable from other tissue types. We note that the preparation of conventional H&E stained slides requires xylene exposure which removes lipids, giving adipose cells a clear appearance. When necessary to stain lipid-containing structures, pathologists routinely use Oil Red O staining. Because DeepDOF-SE examines fresh tissue, the resulting lipid staining pattern is more similar to tissue stained with Oil Red O. Because this is a stain that is commonly used in pathology, it is unlikely that it will result in interpretation challenges; it may be advantageous for evaluating certain tissue types. For example, in breast cancer, the presence of adipose cells is useful for delineating tumor margins. Additionally, residual dyes in excessive phosphate buffer solution (PBS) used to rinse the tissue post-staining may result in residual fluorescence signal; however, as shown in Supplementary Figure 13, the contour from residual dyes is far from the sample and thus does not interfere with tissue imaging.

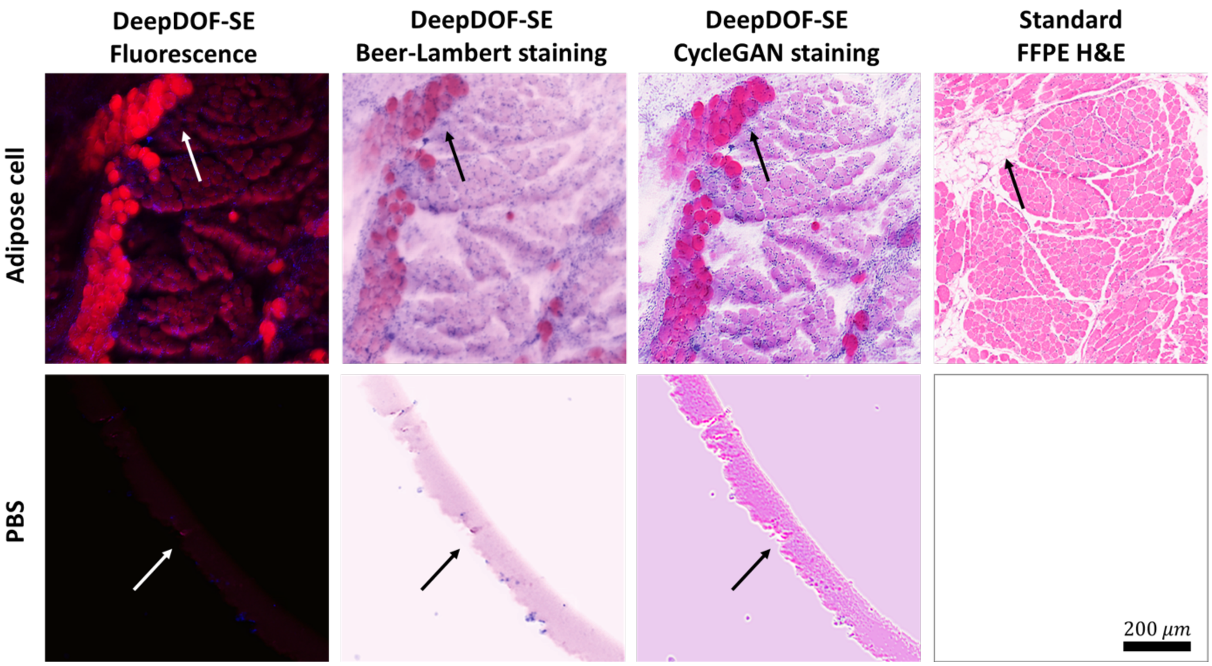

Supplementary Figure 13: Exceptional cases where the CycleGAN virtual staining images do not resemble the standard H&E images. Top row: Adipose cells appear intact in the DeepDOF-SE images, while in the conventional H&E, the cells' cytoplasmic lipids are lost due to H&E sectioning and processing. Bottom row: residual fluorescence from residual rinsing buffer is imaged by DeepDOF-SE, which does not occur in slide-based H&E processing. This artifact is far away from the tissue and can be easily discerned.

Supplementary References:

1. Patel, A. *et al.* Contemporary Whole Slide Imaging Devices and Their Applications within the Modern Pathology Department: A Selected Hardware Review. *J. Pathol. Inform.* **12**, 50 (2021).
2. TKACZYK, E. R. Innovations and Developments in Dermatologic Non-invasive Optical Imaging and Potential Clinical Applications. *Acta Derm. Venereol.* **Suppl 218**, 5–13 (2017).
3. Glaser, A. K. *et al.* Light-sheet microscopy for slide-free non-destructive pathology of large clinical specimens. *Nat. Biomed. Eng.* **1**, 1–10 (2017).
4. Ashman, K. *et al.* A Camera-Assisted Pathology Microscope to Capture the Lost Data in Clinical Glass Slide Diagnosis. 2022.08.31.506042 Preprint at https://doi.org/10.1101/2022.08.31.506042 (2022).
5. Wong, E., Axibal, E. & Brown, M. Mohs Micrographic Surgery. *Facial Plast. Surg. Clin. N. Am.* **27**, 15–34 (2019).
6. Sutton, E. & Hanke, C. W. Microscope Use in Mohs Micrographic Surgery: A Survey of Current and Former Mohs Surgery Fellowship Directors. *Dermatol. Surg. Off. Publ. Am. Soc. Dermatol. Surg. Al* **48**, 786–787 (2022).

7. Patel, Y. G. *et al.* Confocal reflectance mosaicing of basal cell carcinomas in Mohs surgical skin excisions. *J. Biomed. Opt.* **12**, 034027 (2007).
8. Robbins, S. L. (Stanley L. *Robbins & Cotran pathologic basis of disease*. (Elsevier, 2021).
9. Baek, J. Transfer efficiency and depth invariance in computational cameras. in *2010 IEEE International Conference on Computational Photography (ICCP)* 1–8 (IEEE, 2010). doi:10.1109/ICCPHOT.2010.5585098.
10. Stirling, D. R. *et al.* CellProfiler 4: improvements in speed, utility and usability. *BMC Bioinformatics* **22**, 433 (2021).
