# Supplementary material for: DeepDOF-SE: affordable deep-learning microscopy platform for slide-free histology
Source: Nat Commun. 2024 Apr 5;15:2935. doi: 10.1038/s41467-024-47065-2 (PMC10997797; doi:10.1038/s41467-024-47065-2)
Supplement: Supplementary file 2 — Reporting Summary [file 41467_2024_47065_MOESM2_ESM.pdf]

nature portfolio

Corresponding author(s): Ashok Veeraraghavan, Rebecca Richards-Korturm

Last updated by author(s): 2/25/2024

Reporting Summary

Nature Portfolio wishes to improve the reproducibility of the work that we publish. This form provides structure for consistency and transparency in reporting. For further information on Nature Portfolio policies, see our Editorial Policies and the Editorial Policy Checklist.

Statistics

For all statistical analyses, confirm that the following items are present in the figure legend, table legend, main text, or Methods section.

n/a	Confirmed	
☐	☒	The exact sample size (*n*) for each experimental group/condition, given as a discrete number and unit of measurement
☐	☒	A statement on whether measurements were taken from distinct samples or whether the same sample was measured repeatedly
☐	☒	The statistical test(s) used AND whether they are one- or two-sided<br>*Only common tests should be described solely by name; describe more complex techniques in the Methods section.*
☒	☐	A description of all covariates tested
☒	☐	A description of any assumptions or corrections, such as tests of normality and adjustment for multiple comparisons
☐	☒	A full description of the statistical parameters including central tendency (e.g. means) or other basic estimates (e.g. regression coefficient) AND variation (e.g. standard deviation) or associated estimates of uncertainty (e.g. confidence intervals)
☐	☒	For null hypothesis testing, the test statistic (e.g. *F*, *t*, *r*) with confidence intervals, effect sizes, degrees of freedom and *P* value noted<br>*Give P values as exact values whenever suitable.*
☒	☐	For Bayesian analysis, information on the choice of priors and Markov chain Monte Carlo settings
☒	☐	For hierarchical and complex designs, identification of the appropriate level for tests and full reporting of outcomes
☒	☐	Estimates of effect sizes (e.g. Cohen's *d*, Pearson's *r*), indicating how they were calculated

Our web collection on statistics for biologists contains articles on many of the points above.

Software and code

Policy information about availability of computer code

Data collection	The neural networks are trained using custom code written in Python and Tensorflow. The code and relevant dependencies can be found at https://github.com/MJ2695/DeepDOF-SE/. The images are captured using a software API provided by the camera maker (Tucsen). The full sample images are stitched using Microsoft Image Compositor (ver 2.0.3.0).
Data analysis	The image reconstruction and virtual staining is done using custom code written in Python and Tensorflow. The Beer-Lambert virtual staining is done using custom code written in MATLAB.

For manuscripts utilizing custom algorithms or software that are central to the research but not yet described in published literature, software must be made available to editors and reviewers. We strongly encourage code deposition in a community repository (e.g. GitHub). See the Nature Portfolio guidelines for submitting code & software for further information.

Data

Policy information about availability of data

All manuscripts must include a data availability statement. This statement should provide the following information, where applicable:

- Accession codes, unique identifiers, or web links for publicly available datasets
- A description of any restrictions on data availability
- For clinical datasets or third party data, please ensure that the statement adheres to our policy

The training, validation, and testing datasets for the extended depth-of-field network and the imaging data underlying the figures are available at https://zenodo.org/records/10674605. The source data underlying the graphs in this work are provided in the file “DeepDOF-SE_source_data.xlsx”. The training data for the

virtual staining cycleGAN are available under restricted access due to protocol restriction, access can be obtained by contacting the corresponding authors with material transfer agreements.

Research involving human participants, their data, or biological material

Policy information about studies with human participants or human data. See also policy information about sex, gender (identity/presentation), and sexual orientation and race, ethnicity and racism.

Reporting on sex and gender	We did not control or report on the sex or gender of the human participants since this is an ex vivo study. Only the excised tissue was used. While the physicians involved in the protocol has access to the patients' sex and gender, this information was not revealed to the other authors.
Reporting on race, ethnicity, or other socially relevant groupings	We did not control or report on the race, ethnicity, or other socially relevant grouping of the human participants since this is an ex vivo study. Only the excised tissue was used. While the physicians involved in the protocol has access to the patients' race and ethnicity, this information was not revealed to the other authors. Eligible patients were invited to participate in the study without regard to age, race, or ethnicity.
Population characteristics	We did not control for the population of the human participants. Consenting patients undergoing surgery for oral cancer were enrolled.
Recruitment	Subjects with oral squamous carcinoma and oral precancers whose treatment plan includes surgery and are older than 18 will be eligible for enrollment.
Ethics oversight	The study was approved by the Institutional Review Boards at the University of Texas MD Anderson Cancer Center and Rice University.

Note that full information on the approval of the study protocol must also be provided in the manuscript.

Field-specific reporting

Please select the one below that is the best fit for your research. If you are not sure, read the appropriate sections before making your selection.

☒ Life sciences ☐ Behavioural & social sciences ☐ Ecological, evolutionary & environmental sciences

For a reference copy of the document with all sections, see nature.com/documents/nr-reporting-summary-flat.pdf

Life sciences study design

All studies must disclose on these points even when the disclosure is negative.

Sample size	In this study, no statistical method was used to predetermine sample size. For Figure 4 and 5 of the main text, the tissue samples are imaged once with the proposed DeepDOF-SE and once with the conventional baseline. For Figure 6 and 7, the samples are imaged once with the proposed DeepDOF-SE.
Data exclusions	No data was excluded.
Replication	Failure cases were presented in the manuscript supplemental information.
Randomization	No applicable to this study
Blinding	The pathologists interpreting the DeepDOF-SE scans could distinguish them from the standard FFPE slide scans as the appearance differs. They were asked to interpret the DeepDOF-SE scan first, before they were shown the corresponding standard H&E.

Reporting for specific materials, systems and methods

We require information from authors about some types of materials, experimental systems and methods used in many studies. Here, indicate whether each material, system or method listed is relevant to your study. If you are not sure if a list item applies to your research, read the appropriate section before selecting a response.

Materials & experimental systems

n/a	Involved in the study
☒	☐ Antibodies
☒	☐ Eukaryotic cell lines
☒	☐ Palaeontology and archaeology
☒	☐ Animals and other organisms
☒	☐ Clinical data
☒	☐ Dual use research of concern
☒	☐ Plants

Methods

n/a	Involved in the study
☒	☐ ChIP-seq
☒	☐ Flow cytometry
☒	☐ MRI-based neuroimaging

Plants

Seed stocks	*Report on the source of all seed stocks or other plant material used. If applicable, state the seed stock centre and catalogue number. If plant specimens were collected from the field, describe the collection location, date and sampling procedures.*
Novel plant genotypes	*Describe the methods by which all novel plant genotypes were produced. This includes those generated by transgenic approaches, gene editing, chemical/radiation-based mutagenesis and hybridization. For transgenic lines, describe the transformation method, the number of independent lines analyzed and the generation upon which experiments were performed. For gene-edited lines, describe the editor used, the endogenous sequence targeted for editing, the targeting guide RNA sequence (if applicable) and how the editor was applied.*
Authentication	*Describe any authentication procedures for each seed stock used or novel genotype generated. Describe any experiments used to assess the effect of a mutation and, where applicable, how potential secondary effects (e.g. second site T-DNA insertions, mosiacism, off-target gene editing) were examined.*
